# Evaluation of Stresses on Implant, Bone, and Restorative Materials Caused by Different Opposing Arch Materials in Hybrid Prosthetic Restorations Using the All-on-4 Technique

**DOI:** 10.3390/ma14154308

**Published:** 2021-08-01

**Authors:** Feras Haroun, Oguz Ozan

**Affiliations:** Department of Prosthodontics, Faculty of Dentistry, Near East University, Near East Boulevard, Nicosia 99138, Mersin 10, Turkey; oguz.ozan@neu.edu.tr

**Keywords:** All-on-4^®^, hybrid prosthesis, finite element analysis, implant

## Abstract

The long-term success of dental implants is greatly influenced by the use of appropriate materials while applying the “All-on-4” concept in the edentulous jaw. This study aims to evaluate the stress distribution in the “All-on-4” prosthesis across different material combinations using three-dimensional finite element analysis (FEA) and to evaluate which opposing arch material has destructive effects on which prosthetic material while offering certain recommendations to clinicians accordingly. Acrylic and ceramic-based hybrid prosthesis have been modelled on a rehabilitated maxilla using the “All-on-4” protocol. Using different materials and different supports in the opposing arch (natural tooth, and implant/ceramic, and acrylic), a multi-vectorial load has been applied. To measure stresses in bone, maximum and minimum principal stress values were calculated, while Von Mises stress values were obtained for prosthetic materials. Within a single group, the use of an acrylic implant-supported prosthesis as an antagonist to a full arch implant-supported prosthesis yielded lower maximum (Pmax) and minimum (Pmin) principal stresses in cortical bone. Between different groups, maxillary prosthesis with polyetheretherketone as framework material showed the lowest stress values among other maxillary prostheses. The use of rigid materials with higher moduli of elasticity may transfer higher stresses to the peri implant bone. Thus, the use of more flexible materials such as acrylic and polyetheretherketone could result in lower stresses, especially upon atrophic bones.

## 1. Introduction

Due to the current disadvantages of traditional complete dentures, advancing technology and science have been redirected to producing new solutions. Utilizing the current advances in dental implants in conjunction with the All-on-4 treatment concept generally reduces the treatment time, the risk of morbidity, and other possible risks in the edentulous patient. This protocol, which emerged specially to overcome the complicated prosthetic and surgical problems caused by anatomical limitations, has increased its prevalence and has been used frequently [1].

One of the key factors for long-term clinical success is correct planning of the substructure and superstructure materials that support the implant prosthesis. The properties of the material and spatial geometric configuration model of each component have a significant impact on the transference of functional loads and stress distribution in a bone–implant–prosthesis assembly [2]. In the oral environment, these components act in unison with each other, and thus the combination of the materials used is important. Since the nature and magnitude of the intraoral loads are unknown, it is recommended that these stresses are kept to a minimum. Thus, finding suitable dental materials that overcome biomechanical deficiencies and optimize function and aesthetics are desired by many clinicians [3].

A variety of prosthetic materials have been used in the manufacturing of implant-supported fixed full dentures, the most well documented of which is metal-acrylic [4,5]. Long-term follow-up has revealed that this type of restoration is difficult to maintain owing to prosthetic tooth attrition and acrylic fracture [6,7]. With the advent of computer-aided design and computer-aided manufacturing (CAD-CAM) technology, various alternative designs have become possible for implant-supported fixed prosthesis, including Toronto hybrid prosthesis, monolithic zirconia, and porcelain veneered zirconia prosthesis. The Toronto bridge includes the cementation of full veneer restorations on milled titanium or zirconia substructure [8]. As a result, long-term prognosis has been yielded due to superior aesthetics and biomechanics, in addition to the ease of hygienic care [9,10,11,12]. Even though the survival rate is generally high, prosthesis with zirconia substructures can also have a high incidence of minor mechanical complications [13]. Another material that has gained popularity in recent years due to its biocompatibility and favoured physical properties is Polyether-ether-ketone (PEEK) [14]. Veneered PEEK with Polymethylmethacrylates (PMMA) or light-cured composite resins tends to preserve its elastic properties, thus reducing occlusal forces against the restoration in addition to the forces transferred to the restoration, the tooth root, and the opposing dentition [15].

When planning an implant-retained prosthesis, the clinical implication could change drastically depending on the surrounding conditions as intra-oral conditions are not always perfect. While some materials have been found to increase the amount of stress transmitted to the opposing prosthesis, other materials have been shown to transfer lower stresses [3,7,16,17,18]. As a result, it has been believed that the type of opposing structure influences the level of crestal bone change and the amount of stresses that are being transferred [19].

Appropriate selection of implant and prosthetic materials is very important for the longevity, stability, and proper function of the implant-supported prosthesis. In the field of dentistry, the use of finite element analysis has gained its popularity due to its ability to provide useful information about stress values and patterns in the “All-on-4” concept [20]. Using three-dimensional finite element analysis, this study aims to examine the stress distribution in the maxillary “All-on-4” prosthesis across different material combinations to offer certain recommendations to clinicians.

## 2. Materials and Methods

A 3D model of an edentulous maxilla was built using data collected from the Visible Human Project (US National Library of Medicine, Bethesda, MD, USA). Utilizing the software programs VRMESH version 6.1 (Bellevue, WA, USA) and Rhinoceros 4.0 (Robert McNeel & Associates, Seattle, WA, USA), the geometry model was modified with a 2 mm cancellous bone layer around the spongious bone with 2 mm-thick mucosa. An optical scanner (SmartOptics 3D scanner, Bochum, Germany) was used to scan implants and prosthetic components within a 10 μm accuracy ratio, and data were reconstructed with VRMESH software in conjunction with Rhinoceros 4.0 to model the structures.

Anteriorly, the vertical implants were modelled in the lateral incisal area based on the dimensions of a NobelActive RP implants (Nobel Biocare, Zurich, Switzerland) (with a diameter of 4.3 mm and a length of 13 mm). Posteriorly, standard length implants were modelled at an angle of 30° and placed into the second premolar region. Anterior implants were fitted with straight multi-unit abutments while posterior implants were fitted with angled multi-unit abutments (Nobel Biocare, Sweden).

**Figure 1:**
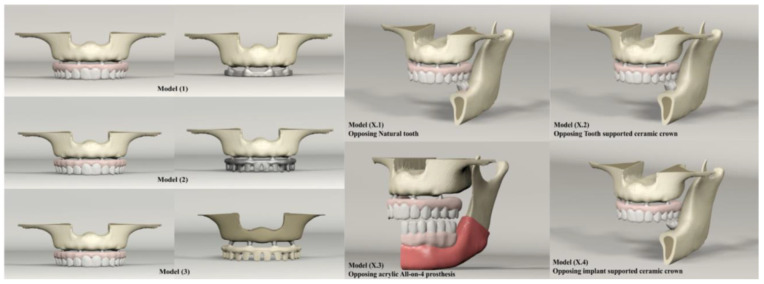
Maxillary and mandibular models used in the study.

According to superstructure material and framework design, three main models were created in the maxillary jaw. For analysis, each maxillary model was opposed by four different models in the lower jaw to simulate different loading scenarios (Table 1 and Figure 1). The materials used in this study were considered to be homogeneous, linearly elastic, and isotropic. The characteristics of the materials used in our study are shown in (Table 2).

**Figure 2:**
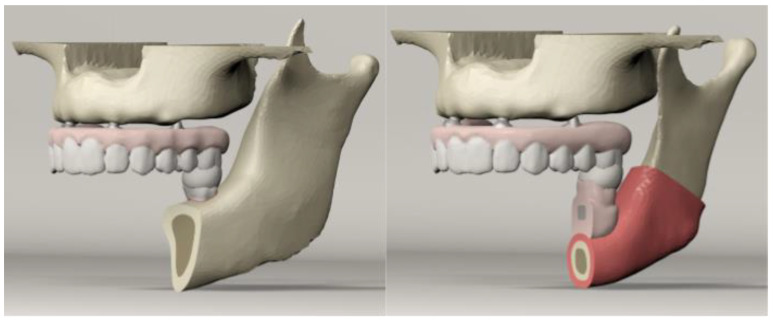
Spherical solid material simulating foodstuff located on the left first molar region.

For each model, an occlusal load was delivered to the left first molar area using a spherical solid substance (12 mm in diameter) that simulated foodstuff (Figure 2). To closely simulate the forces exerted while chewing, weighting factors have been assigned to each muscle of the primary masticatory muscles for the five clenching tasks [21] (Table 3).

**Figure 3:**
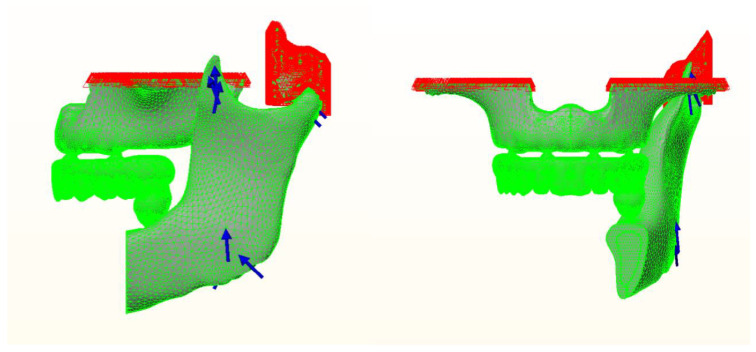
Boundary conditions and force directions used in the analysis.

To avoid displacement, the midsagittal, posterior, and top cutting planes were constrained in all three directions x, y, and z (Figure 3). Complete osseointegration between Implant and bone surfaces was presumed. All the bodies were assumed to be perfectly bonded together through the contact surfaces with no relative movement along their entire interfaces.

The maximum principal stress (Pmax) and minimum principal stress (Pmin) stresses were computed for the cortical bone. Prosthetic components and implants were considered as ductile materials and had their Von Mises stress values calculated.

## 3. Results

### 3.1. Stresses in Peri-Implant Cortical Bone

The stress distribution in the cortical bone around the posterior implant region of the loaded side is illustrated in (Figure 4, Figure 5, Figure 6, Figure 7, Figure 8, Figure 9, Figure 10 and Figure 11).

#### 3.1.1. Maximum Principal Stresses (Pmax)

For all three groups, the lowest value (23.2 N/mm^2^) was recorded in group 3 (PEEK and Zirconia), followed by group 1 (Titanium and PMMA) (32.2 N/mm^2^) and group 2 (Titanium and Zirconia) (34.3 N/mm^2^), respectively, all when opposed by a mandibular acrylic All-on-4 with titanium framework prosthesis in the lower jaw. The highest value (72.7 N/mm^2^) was found in group 2 (Titanium and Zirconia) and group 3 (PEEK and Zirconia) (68.7 N/mm^2^) when opposed by an implant-supported full ceramic crown Followed by group 2 (Titanium and Zirconia) (65.7 N/mm^2^) when opposed by tooth-supported full ceramic crown. Opposing the prosthesis by a natural tooth or a tooth-supported ceramic crown did not show major difference in results across all groups.

#### 3.1.2. Minimum Principal Stresses (Pmin)

A similar stress pattern has been observed across all the groups. The lowest values (-39.5 N/mm^2^) were found to be when group 3 (PEEK and Zirconia) models were opposed by acrylic All-on-4 with titanium framework, followed by group 1 (Titanium and PMMA) then group 2 (Titanium and Zirconia) respectively. The highest value (−156.2 N/mm^2^) was observed in group 2 (Titanium and Zirconia) opposing an implant-supported full ceramic crown in the lower jaw, followed by tooth-supported full ceramic crown (−146.8 N/mm^2^) of the same group.

### 3.2. Stresses in Implants

**Figure 12:**
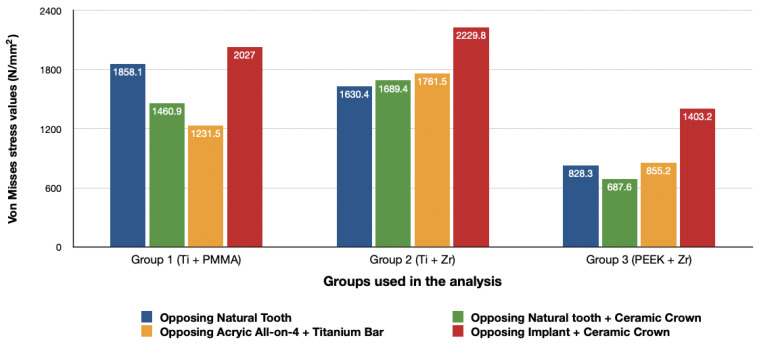
Von Mises stress values on posterior implants under loads.

**Figure 13:**
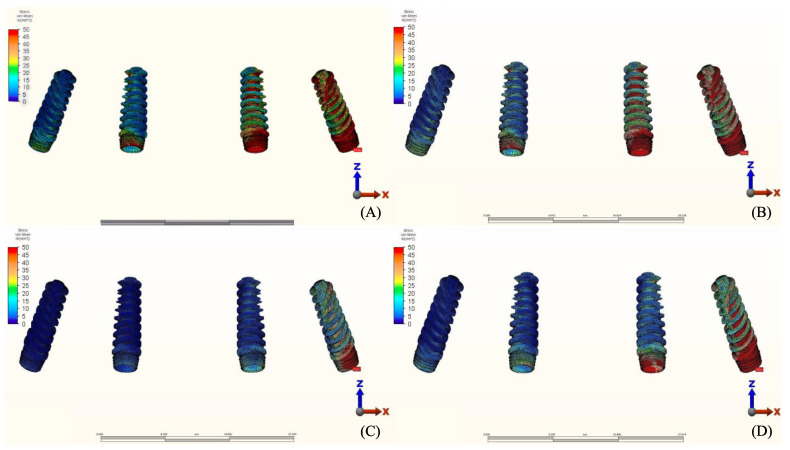
Implant stress distribution for group 1, titanium bar with acrylic teeth. (**A**) Model 1.1, opposing natural tooth. (**B**) Model 1.2, opposing tooth-supported full ceramic crown. (**C**) Model 1.3, opposing acrylic prosthesis with titanium framework. (**D**) Model 1.4, opposing implant-supported full ceramic crown.

**Figure 14:**
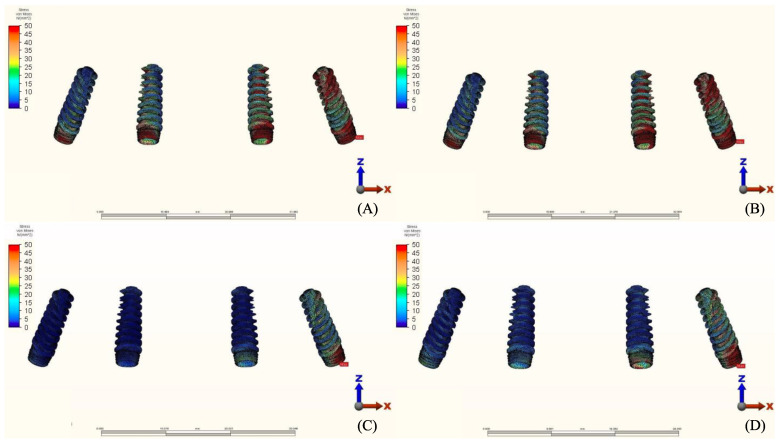
Implant stress distribution for group 2, titanium bar with resin composite gingiva and ceramic superstructure with zirconium (Toronto bridge). (**A**) Model 2.1, opposing natural tooth. (**B**) Model 2.2, opposing tooth-supported full ceramic crown. (**C**) Model 2.3, opposing acrylic prosthesis with titanium framework. (**D**) Model 2.4, opposing implant-supported full ceramic crown.

**Figure 15:**
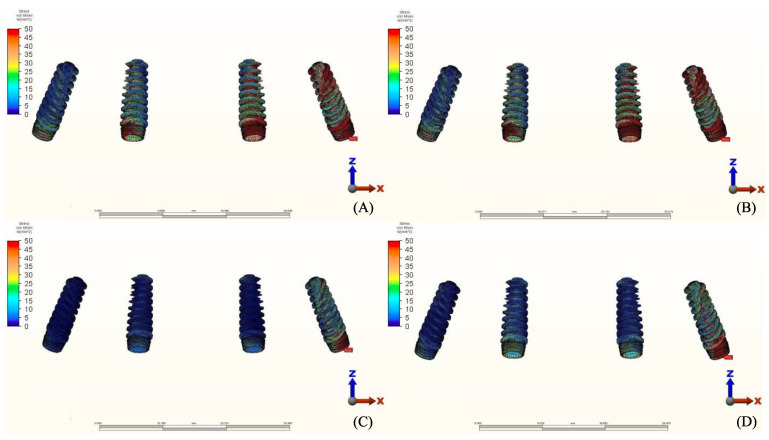
Implant stress distribution for group 3, PEEK bar with composite resin gingiva and ceramic superstructure with zirconium (Toronto bridge). (**A**) Model 3.1, opposing natural tooth. (**B**) Model 3.2, opposing tooth-supported full ceramic crown. (**C**) Model 3.3, opposing acrylic prosthesis with Ti framework. (**D**) Model 3.4, opposing implant-supported full ceramic crown.

Maximum equivalent stress values and their distribution in the implants of each model are illustrated in Figure 12, Figure 13, Figure 14 and Figure 15. Within each group, maxillary prostheses opposed by implant-supported full ceramic crown registered the highest stress values in the implant body mesially near the neck of the implant across all groups. The highest value (2229.8 N/mm^2^) was found in group 2 (Titanium and Zirconia) followed by group 1 (Titanium and PMMA) (2027 N/mm^2^). The lowest value (687.6 N/mm^2^) was found in group 3 (PEEK and Zirconia) when opposed by the tooth-supported ceramic crown followed by natural tooth as an antagonist (828.3 N/mm^2^), and when opposed by an acrylic All-on-4 with titanium framework (855.2 N/mm^2^), all within the same group.

### 3.3. Stresses in Framework

**Figure 16:**
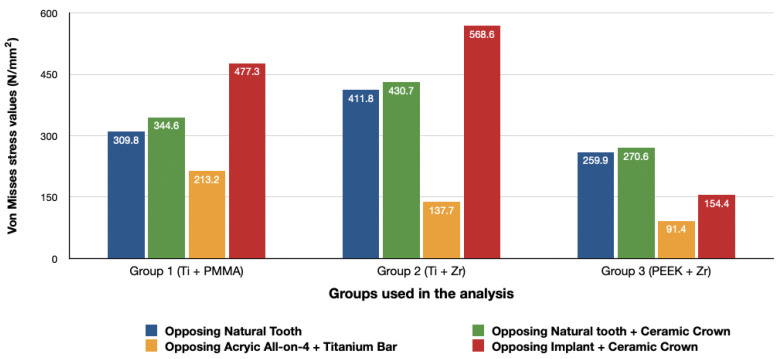
Values of Von Mises stresses on Framework under loads.

**Figure 17:**
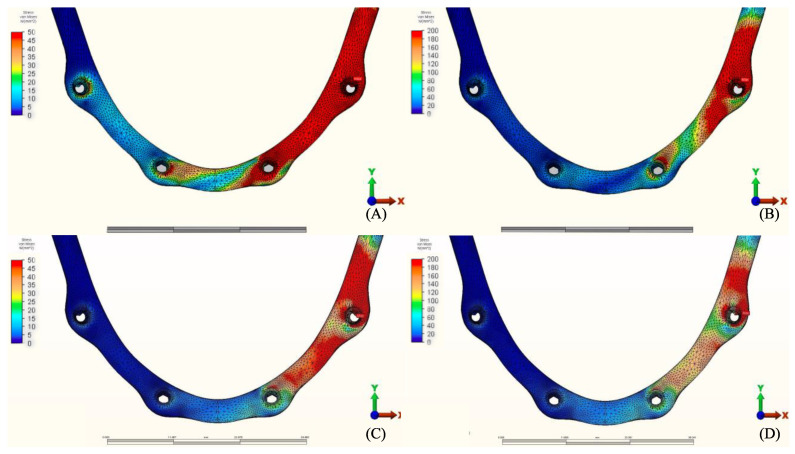
Stress distribution in the framework for group 1, titanium bar with acrylic teeth. (**A**) Model 1.1, opposing natural tooth. (**B**) Model 1.2, opposing tooth-supported full ceramic crown. (**C**) Model 1.3, opposing acrylic prosthesis with titanium framework. (**D**) Model 1.4, opposing implant-supported full ceramic crown.

**Figure 18:**
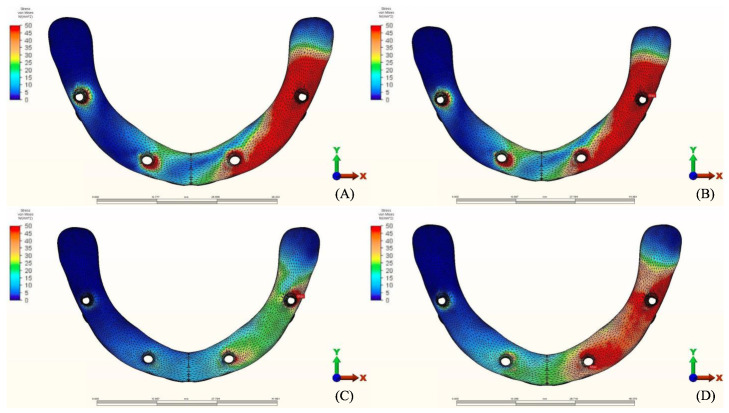
Stress distribution in the framework for group 2, titanium bar with resin composite gingiva and ceramic superstructure with zirconium (Toronto bridge). (**A**) Model 2.1, opposing natural tooth. (**B**) Model 2.2, opposing tooth-supported full ceramic crown. (**C**) Model 2.3, opposing acrylic prosthesis with titanium framework. (**D**) Model 2.4, opposing implant-supported full ceramic crown.

**Figure 19:**
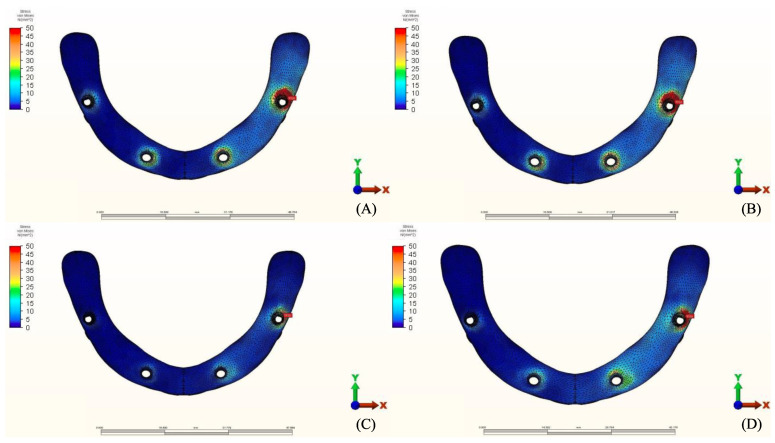
Stress distribution in the framework for group 3, PEEK bar with composite resin gingiva and ceramic superstructure with zirconium (Toronto bridge). (**A**) Model 3.1, opposing natural tooth. (**B**) Model 3.2, opposing tooth-supported full ceramic crown. (**C**) Model 3.3, opposing acrylic prosthesis with titanium framework. (**D**) Model 3.4, opposing implant-supported full ceramic crown.

The von Mises stresses in the framework and stress distribution for each model are shown in Figure 16, Figure 17, Figure 18 and Figure 19. Generally, the PEEK framework showed the lowest stress values when compared to titanium. PEEK framework opposed by an acrylic All-on-4 prosthesis in the mandible registered the lowest value (91.4 N/mm^2^) followed by group 2 (Titanium and Zirconia) (137.7 N/mm^2^) when opposed by an acrylic All-on-4 prosthesis followed by group 3 (PEEK and Zirconia) (154.4 N/mm^2^) when opposed by an implant supported ceramic crown in the lower jaw. The highest stress value was found in the titanium framework of group 2 (Titanium and Zirconia) (568.6 N/mm^2^) followed by group 1 (Titanium and PMMA), both when the maxillary jaw was opposed by an implant-supported full ceramic crown.

## 4. Discussion

The recent advances in CAD/CAM technology over the last decade have enabled clinicians to use different material combinations. Research showing the effect of different prosthetic material on stress distribution in implant-supported complete arch prosthesis is well documented; however, the number of researches discussing the effect of the opposing arch material is close to none. In this study, FEA has been utilized to mechanically evaluate the response of different prosthetic designs with different material combinations for manufacturing an implant-supported full-arch dental prosthesis opposing different materials and various abutments (tooth/implant).

The evolution of the biomechanical properties of the bone-implant interface is key to the surgical success of implant procedures. Studies have shown that functional loading of the implant aids in the formation of bone [22]. On the other hand, mechanical overloading could result in the failure of the implant in conjunction with host-related problems [23]. Bone loss during the first year of the implant could be attributed to the surgical procedures; however, in the following years it could be caused by immunological, surgical, or prosthetic reasons [24]. It has been found that supra-occlusal contacts significantly increased bone resorption in the presence of inflammation response in the peri-implant bone [25]. Thus, it has been suggested to keep these occlusal stresses within the biological boundaries of the human bone.

Chewing forces are undeniably transmitted to the restoration, and these forces do not diminish but rather change their form, which is disseminated throughout the restoration-implant complex. Restorative materials, cement layer, abutment, screw, implants, and peri-implant bone might all receive the energy of the chewing force. It has been suggested by several authors that the use of rigid materials could result in a better distribution of stresses [3,16,26,27]. However, in our study, the use of acrylic in combination with titanium showed fairly low stresses in the bone localized around the implant when compared to zirconium. In addition to that, all the maxillary prosthesis showed lower stresses in the bone surrounding the implant when opposed by a mandibular implant-supported acrylic prosthesis. These results correlate to the result obtained by other authors [18,28,29]. Another study showed that the use of acrylic resins resulted in a lower stress distribution when compared to ceramics [17]. This might be caused by the acrylic resin’s modulus of elasticity (2.2 GPa) which is lower than both the ceramic (82 GPa) and zirconia (210 GPa), which enables greater absorption and lower stresses transference to the supporting bone. [28,29,30]. In a similar study conducted by Elsayed et al. [31], it has been shown that stresses were lower in the peri-implant bone when the prosthesis was opposed by acrylic denture in comparison to natural teeth. In our study, using an implant-supported ceramic crown as an antagonist resulted in the highest stress values among all the groups. These findings have been confirmed by various in vivo research by emphasizing the increased presence of mechanical complications when the prosthesis is opposed by an implant-supported crown [9,32,33,34,35]. In addition, authors have noticed lower bone loss around the implant (0.2 mm) in instances with natural tooth and higher bone loss (0.6 mm) when it is opposed by an implant restoration at the same time [19]. These findings could be attributed to the lack of proprioception by the patient and/or the lack of shock-absorbing capacity by the prosthesis or the lack of periodontal ligament which aids in stress release during function [9,36,37]. Natural teeth in occlusion with an implant would experience a slight intrusion during function but the same amount of vertical/lateral displacement will not be observed for the implant [38]. The rigid bone-implant interface and the lack of stress releasing implant components does not provide physioelasticity and are incapable of detecting forces such as teeth [39].

Framework material has been thought to affect the amount of stresses transferred to surrounding components by some of the authors [16,40,41], while others stated that it has no significant effect [42]. Studies comparing PEEK to titanium and zirconia showed higher stress concentration within the framework in stiffer materials such as zirconia, followed by titanium [16,40]. Despite the increased stresses in zirconia frameworks, some authors have claimed that increased stiffness of the frameworks may help in transferring lower loads to the implant and prosthetic components than less rigid ones, avoiding prosthesis failure [16,27,43]. In our study, the use of PEEK yielded a reduction in the stresses located in the framework followed by titanium with acrylic and titanium with zirconia respectively. Our results concur with the findings of Lee et al. [43]. Sirandony et al. [44] also reported lower stress values in the framework when using PEEK material but with higher stresses in bone. The difference in the results when comparing it with our study could be due to the fact that the authors tested the framework material only without the use of any prosthetic material over the framework. The use of superstructure material in combination with the framework materials could act as a stress dampener hence lower stress values in the bone observed when using PEEK in comparison to titanium frameworks in our study.

Due to the irregular nature of the biological materials, finite element analysis (FEA) has been proved to be a valid approach for assessing the mechanical behaviour of materials in the oral cavity in the field of dentistry [45]. FEA gives the ability to visualize structures that are superimposed and it allows the establishment of the location, magnitude, and direction of an applied force at any given point. Since FEA does not alter the physical properties of the material it is also easily repeatable [46,47]. Intraoral forces are known to be multi vectorial and differ in value in relation to their direction [48]. It has been noticed that the use of implant-supported full arch prosthesis affects the thickness of the masseter muscle, chewing efficiency, and biting force [49]. Similar studies have used alternating force vectors (vertical and horizontal) with fixed loadings directed to the tubercle [16,50]. However, in our study, the five muscles of clenching have been modeled and each muscle has been given a corresponding weighting factor in order to simulate intra-oral forces as closely as possible [21]. It is worth mentioning that selecting the proper occlusion scheme and making the necessary occlusal modifications and adjustments have a significant impact on the amount of forces generated in the prosthesis’ components. [1,51,52]. Due to the complexity of adjusting the occlusion in each model and to standardize the process in our study, the use of a spherical solid object that contacts the cusps of the posterior molars has been suggested. Moreover, it guarantees the exertion of forces in vertical, horizontal, and oblique directions [53]. The models analyzed using FEA are considered to be homogeneous, isotropic, and linear. It is important to consider these restrictions when elucidating the results as oral tissues are more complex and aniscopic. In addition, these experiments were made at the in-vitro level, and therefore, the actual representation of osseointegration and functions of the periodontal ligament could not be simulated. Because the assumption of full osseointegration may not exist under actual clinical settings, finite element analysis may not entirely mimic the true clinical scenario. Although our study followed the classic All-on-4 configuration by tilting posterior implants to a 30° angle, changing the angle or the configuration of the implants could result in different results from a biomechanical standpoint. Due to the rapid advancements in dental materials technology, long-term in-vivo studies are necessary. New clinical studies with variable implant number such as six or eight implants, different implant configurations and sizes, and different prosthetic material combinations can support this study and provide better understanding of the bone behavior in the future.

## 5. Conclusions

Within the scope of this research, there are certain limitations; however, the following has been concluded:

(1). The use of materials with a low modulus of elasticity such as acrylic and PEEK could reduce the amount of stresses transmitted to the bone.
(2). The use of implant-supported ceramic prosthesis as an antagonist to another implant-supported full arch prosthesis increases the amount of stresses transmitted to the bone.
(3). There is no difference in the amount of stresses transmitted when comparing natural tooth to tooth-supported ceramic restorations as an antagonist for a full arch implant-supported prosthesis.

## Figures and Tables

**Figure 4:**
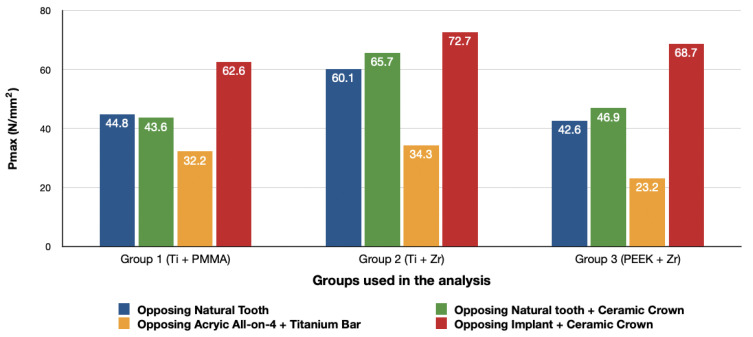
Maximum principal stresses (N/mm^2^) on cortical bone.

**Figure 5:**
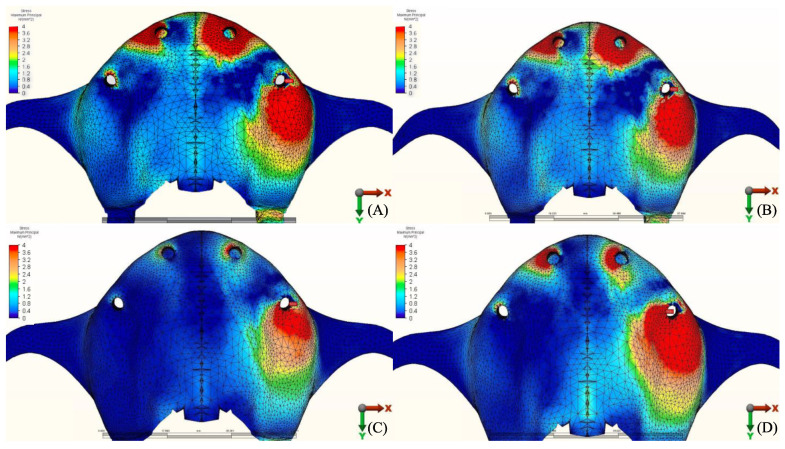
Pmax stress distribution in cortical bone for group 1, titanium bar with acrylic teeth. (**A**) Model 1.1, opposing natural tooth. (**B**) Model 1.2, opposing tooth-supported full ceramic crown. (**C**) Model 1.3, opposing acrylic prosthesis with titanium framework. (**D**) Model 1.4, opposing implant-supported full ceramic crown.

**Figure 6:**
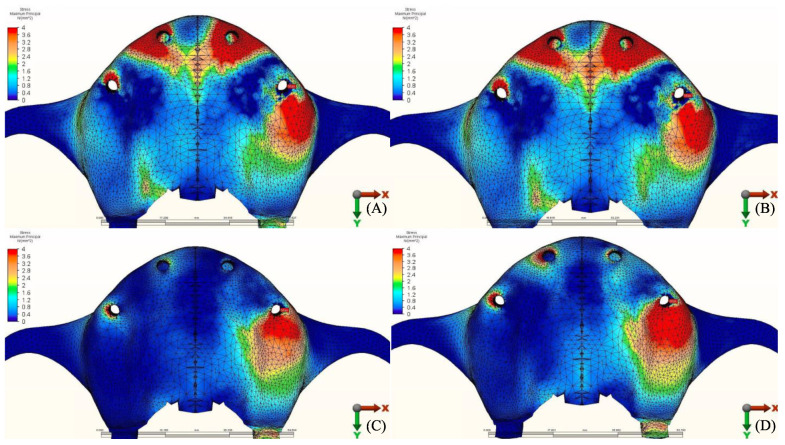
Pmax stress distribution in cortical bone for group 2, titanium bar with resin composite gingiva and ceramic superstructure with zirconium (Toronto bridge). (**A**) Model 2.1, opposing natural tooth. (**B**) Model 2.2, opposing tooth-supported full ceramic crown. (**C**) Model 2.3, opposing acrylic prosthesis with titanium framework. (**D**) Model 2.4, opposing implant-supported full ceramic crown.

**Figure 7:**
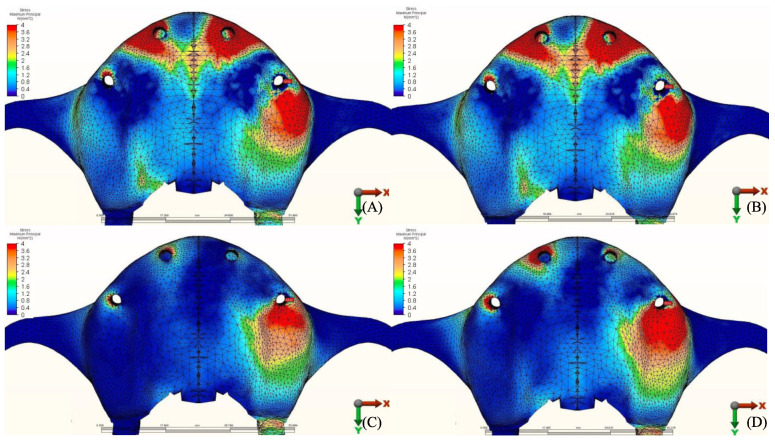
Pmax stress distribution in cortical bone for group 3, PEEK bar with composite resin gingiva and ceramic superstructure with zirconium (Toronto bridge). (**A**) Model 3.1, opposing natural tooth. (**B**) Model 3.2, opposing tooth-supported full ceramic crown. (**C**) Model 3.3, opposing acrylic prosthesis with titanium framework. (**D**) Model 3.4, opposing implant-supported full ceramic crown.

**Figure 8:**
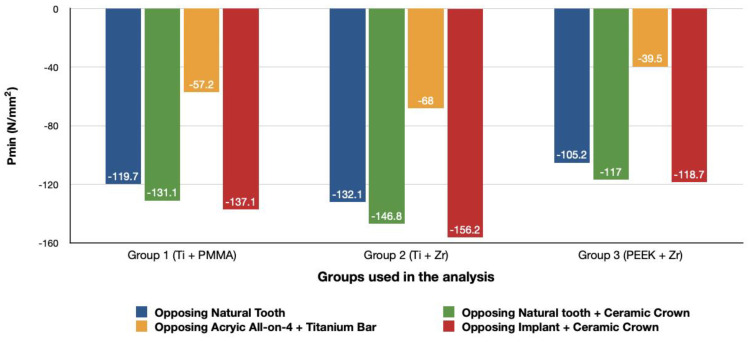
Minimum principal stresses (N/mm^2^) on cortical bone.

**Figure 9:**
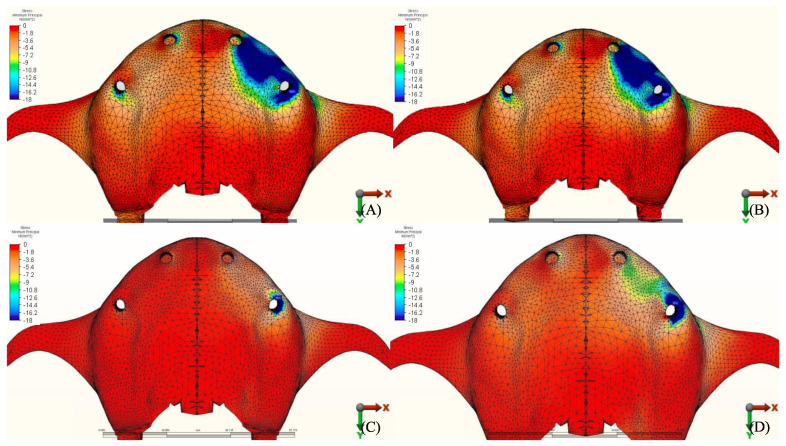
Pmin stress distribution in cortical bone for group 1, titanium bar with acrylic teeth. (**A**) Model 1.1, opposing natural tooth. (**B**) Model 1.2, opposing tooth-supported full ceramic crown. (**C**) Model 1.3, opposing acrylic prosthesis with titanium framework. (**D**) Model 1.4, opposing implant-supported full ceramic crown.

**Figure 10:**
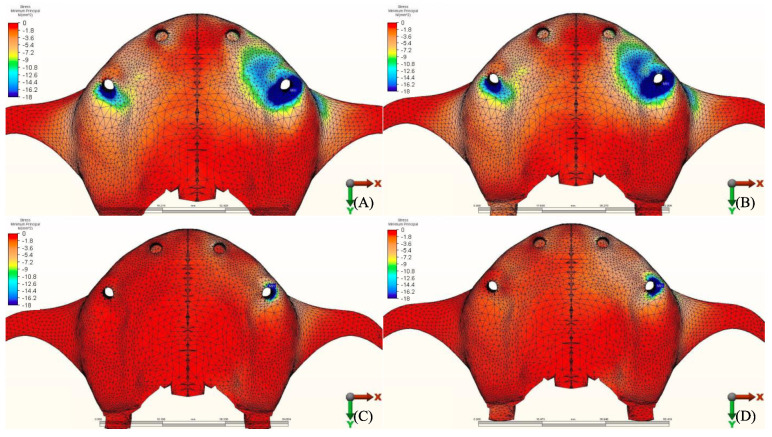
Pmin stress distribution in cortical bone for group 2, titanium bar with resin composite gingiva and ceramic superstructure with zirconium (Toronto bridge). (**A**) Model 2.1, opposing natural tooth. (**B**) Model 2.2, opposing tooth-supported full ceramic crown. (**C**) Model 2.3, opposing acrylic prosthesis with titanium framework. (**D**) Model 2.4, opposing implant-supported full ceramic crown.

**Figure 11:**
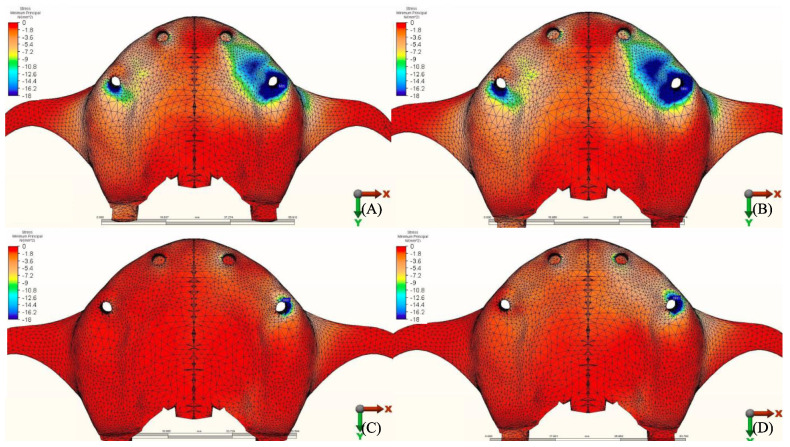
Pmin stress distribution in cortical bone for group 3, PEEK bar with composite resin gingiva and ceramic superstructure with zirconium (Toronto bridge). (**A**) Model 3.1, opposing natural tooth. (**B**) Model 3.2, opposing tooth-supported full ceramic crown. (**C**) Model 3.3, opposing acrylic prosthesis with titanium framework. (**D**) Model 3.4, opposing implant-supported full ceramic crown.

**Table 1:** Element and node numbers of maxillary and mandibular models.

Maxilla	Mandible	Number of Elements	Number of Nodes
Model 1: Titanium bar with acrylic teeth	1.1 Natural tooth	1,173,283	265,982
	1.2 Full ceramic crown	1,214,741	279,924
	1.3 Acrylic All-on-4	1,502,434	365,627
	1.4 Implant-supported ceramic crown	1,347,938	299,558
Model 2: Titanium bar with resin composite gingiva and ceramic superstructure with zirconium (Toronto bridge)	2.1 Natural tooth	1,256,911	283,379
	2.2 Full ceramic crown	1,298,424	297,321
	2.3 Acrylic All-on-4	1,586,062	383,002
	2.4 Implant-supported ceramic crown	1,431,626	316,955
Model 3: PEEK bar with composite resin gingiva and ceramic superstructure with zirconium (Toronto bridge)	3.1 Natural tooth	1,256,927	283,379
	3.2 Full ceramic crown	1,298,373	297,321
	3.3 Acrylic All-on-4	1,586,062	383,002
	3.4 Implant-supported ceramic crown	1,431,566	316,955

**Table 2:** Mechanical Properties of the Materials.

Material	Elastic Modulus	Poisson Ratio
Tempro-mandibular disk	44.1	0.4
Cortical bone	13,700	0.3
Spongy bone	1370	0.3
Periodontal ligament	68.9	0.45
Mucosa	1	0.37
Dentin	18,600	0.32
Enamel	84,100	0.33
Acrylic resin	2200	0.31
Titanium (Grade 4)	105,000	0.37
Titanium (Grade 5)	114,000	0.33
Zircon	210,000	0.3
Poly-Ether Ether Keton (PEEK)	4100	0.4
Composite (Gradia)	50,000	0.3
Feldspathic ceramic	82,800	0.35
Food stuff	84.1	0.33

**Table 3:** Node number and weighting factor allocated to the masticatory muscles responsible for the five clenching tasks.

Muscles	Node Number	Weighting Factor (Newton)
	Right	
Superficial masseter	67	190.4
Deep masseter	38	81.6
Medial pterygoid	51	174.8
Anterior temporalis	43	158
Middle temporalis	18	95.6
Posterior temporalis	15	75.6
Inferior lateral pterygoid	5	66.9
Superior lateral pterygoid	4	28,7
Anterior digastric	8	40

## Data Availability

Not applicable.
